# Radical Resection Using the Commando Procedure for Recurrent Cardiac Intimal Sarcoma in a Young Adult: A Case Report

**DOI:** 10.5761/atcs.cr.26-00101

**Published:** 2026-06-04

**Authors:** Masahide Komagamine, Yoshiki Yamasaki, Tsuyoshi Kono, Masahiro Tomita, Satoshi Kinebuchi, Daijun Tomimoto, Kan Nawata

**Affiliations:** Department of Cardiovascular Surgery, St. Marianna University School of Medicine, Kawasaki, Kanagawa, Japan

**Keywords:** cardiac intimal sarcoma, recurrent cardiac tumor, commando operation, Manouguian procedure

## Abstract

Primary cardiac intimal sarcoma is an extremely rare, high-grade malignancy characterized by rapid progression and high early recurrence rates. We report the case of a 28-year-old male who underwent emergency resection for a large left atrial intimal sarcoma (10 × 8 cm). Follow-up imaging at 6 months revealed a 15-mm recurrent tumor infiltrating the left atrial septum, despite an initially stable recovery. We prioritized radical resection over surgical risk, given the patient’s youth and the aggressive behavior of the tumor. Therefore, a “Commando” procedure consisting of radical tumor resection, double valve replacement, and reconstruction of the intervalvular fibrosa using the Manouguian technique was performed. Histopathological examination confirmed recurrent intimal sarcoma with negative surgical margins. Despite its high invasiveness, the Commando procedure is viable for primary treatment for recurrent cardiac intimal sarcomas in young patients. Aggressive surgical reconstruction remains the only viable option to improve the prognosis of this otherwise fatal malignancy.

## Introduction

Primary cardiac tumors are rare and have an autopsy-based incidence of 0.001%–0.030%.^1)^ Most of them (75%) are benign, and the most prevalent type is myxoma. Malignancies account for approximately 25% of cases, and sarcomas are the most common histological subtype.^2)^

Intimal sarcomas frequently arise from the vasculature, such as the aorta and pulmonary artery. Primary cardiac intimal sarcomas are characterized by high-grade malignant potential and extreme rarity, and only a handful of cases have been reported to date.

## Case Report

**Fig. 1:**
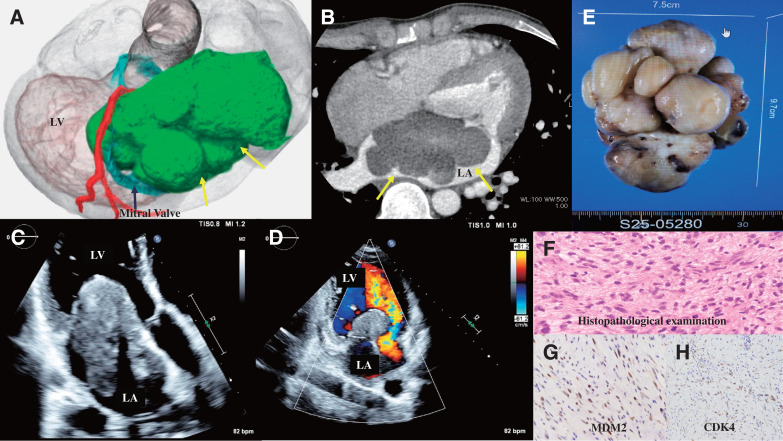
(**A**, **B**) A chest 3D CT revealed a large tumor (yellow arrows) measuring approximately 10 cm in diameter and almost completely filling the LA. (**C**, **D**) TTE showing the giant left atrial tumor obstructing the mitral valve. (**E**) Gross pathological findings show a multinodular, fused tumor measuring 9.7 × 7.5 cm and having a broad-based attachment. (**F**) Histopathological examination revealed the proliferation of neoplastic cells with nuclear atypia and eosinophilic cytoplasm. (**G**, **H**) Immunohistochemistry revealed that the tumor cells were immunoreactive for MDM2 and CDK4. 3D CT, 3-dimensional computed tomography; LA, left atrium; RA, right atrium; TTE, transthoracic echocardiogram

A 28-year-old male presented with a 1-month history of exertional dyspnea and palpitations. He was transported to our emergency department following a syncopal episode. Chest 3-dimensional computed tomography revealed a large tumor with a dimension of approximately 10 × 8 cm, almost completely filling the left atrium (**Figs. 1A** and **1B**). Transthoracic echocardiography showed that the large left atrial tumor was obstructing the mitral valve (**Figs. 1C** and **1D**). The patient was hemodynamically unstable with a pressure gradient of 14 mmHg across the mitral valve. Emergency surgery was performed due to the high risk of sudden cardiac arrest.

The tumor in the left atrium was firm, solid, and a broad-based attachment to the left atrial endocardium and the anterior leaflet of the mitral valve. It was carefully dissected using electrocautery and sharp dissection and was successfully resected *en bloc*.

Gross pathological findings showed a multinodular, fused tumor measuring 9.7 × 7.5 cm that had a broad-based attachment (**Fig. 1E**). Histopathological examination revealed the proliferation of neoplastic cells with nuclear atypia and eosinophilic cytoplasm (**Fig. 1F**). Immunohistochemistry revealed that the tumor cells were immunoreactive for MDM2 and CDK4, which supported the final diagnosis of intimal sarcoma (**Figs. 1G** and **1H**).

**Fig. 2:**
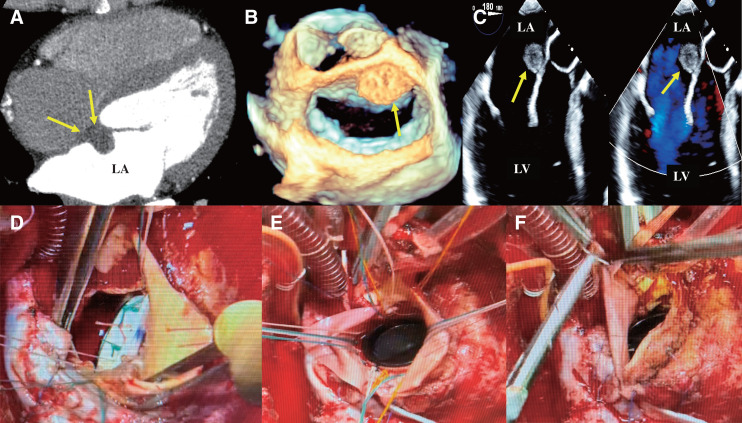
(**A**, **B**) A chest 3D CT. (**C**) A TTE revealed a 15-mm recurrent tumor infiltrating (yellow arrows) the left atrial septum and aortic annulus. (**D**–**F**) Intraoperative photographs for the “Commando” procedure using the Manouguian technique. (**D**) Mitral valve replacement with 25/33-mm On-X mechanical heart valve. (**E**) Aortic valve replacement with a 23-mm SJM Regent mechanical valve. (**F**) Anterior aortic incision was closed using the bovine pericardial patch. 3D CT, 3-dimensional computed tomography; LA, left atrium; RA, right atrium; TTE, transthoracic echocardiogram

Despite an initially stable recovery, follow-up imaging at 6 months revealed a 15-mm recurrence in the left atrial septum, with findings suggestive of infiltration toward the aortic annulus (**Figs. 2A**–**2C**). Given the young age of the patient and aggressive nature of the tumor, we prioritized radicality over surgical risk. A “Commando” procedure consisting of radical tumor resection, double valve replacement, and reconstruction of the intervalvular fibrosa using the Manouguian technique was performed. To address the dense mediastinal adhesions from the previous sternotomy, a cardiopulmonary bypass was established via the right femoral artery and vein. Following redo sternotomy, the Manouguian technique was employed, extending the incision from the commissure between the left coronary cusp and the noncoronary cusp toward the anterior mitral leaflet (AML).

The tumor had a broad attachment to the left atrial intima, extending near the mitral annulus. The tumor, along with its attachment site and surrounding tissues, was resected en bloc. Intraoperative frozen section analysis confirmed the diagnosis of intimal sarcoma with negative surgical margins. Cryoablation (CryoICE; AtriCure Inc., Mason, OH, USA) was applied to the resection margins and surrounding tissues.

The AML was resected while the posterior leaflet was preserved, and a 25/33-mm mechanical heart valve (On-X Life Technologies, Inc., Austin, TX, USA; distributed by Senko Medical Instrument Mfg. Co., Ltd., Tokyo, Japan) was implanted (**Fig. 2D**). A bovine pericardial patch (Edwards Lifesciences, Irvine, CA, USA) was used to reconstruct the left atrium by suturing the AML side to the roof of the atrium. Aortic valve replacement was performed using a 23-mm SJM Reagent mechanical valve (Abbott, St. Paul, MN, USA) (**Fig. 2E**). Finally, the anterior aortic incision was closed by extending the bovine pericardial patch used for the atrial wall reconstruction (**Fig. 2F**).

Definitive histopathological analysis confirmed a complete oncological resection (R0) of the recurrent intimal sarcoma. Microscopic tumor-free margins were successfully achieved across all resected sections, specifically encompassing the aortic annulus, the lateral margin of the left atrial septum, and the mitral valve apparatus following total excision of the AML.

Although the postoperative course required an extended period for recovery, the patient underwent intensive rehabilitation and was transferred on postoperative day 50 with no evidence of residual tumor.

## Discussion

In clinical practice, myxoma is the most strongly suspected when a neoplastic lesion is encountered in the left atrium of a young patient. Myxomas account for approximately half of all benign cardiac tumors. They comprise 75% of primary cardiac tumors and typically occur in males aged 30–50 years. Myxomas, lipomas, and thrombi are common differentials in the left atrium; sarcomas must also be considered, although they are rare.^3)^ Preoperative echocardiographic findings of myxomas often reveal an isoechogenic mass with a narrow stalk, whereas sarcomas typically present as a broad-based mass with heterogeneous echogenicity.

Primary cardiac sarcoma is an extremely rare malignancy that can arise in any part of the heart without sex predilection.^4)^ The most common subtypes are angiosarcoma (37%), undifferentiated sarcoma (24%), and malignant fibrous histiocytoma (11%–24%).^5)^ Intimal sarcomas are mesenchymal neoplasms that arise predominantly in the great vessels but also occur within the cardiac chambers or on valves.^6)^ Cardiac intimal sarcoma is a highly aggressive malignancy, with a reported median survival of 3–12 months after diagnosis.^4)^ Complete surgical resection is currently the only treatment known to improve prognosis. A study of 34 cases at the Mayo Clinic showed a median survival of 17 months with complete resection relative to only 5 months with incomplete resection.^7)^ Similarly, Isambert et al., from the French Sarcoma Group, reported a median overall survival (OS) of 17.2 months. For their cohort, surgery was performed for 65% of patients, and OS was significantly longer for complete resection (38.8 months) relative to incomplete resection (18.2 months) or no resection (11.2 months). Chemotherapy was associated with improved OS only for patients who did not undergo surgery.^8)^ Some reports suggest that combining surgery with postoperative radiotherapy may reduce the frequency of metastasis.^9)^

In the present case, emergency surgery was performed immediately upon admission due to the history of syncope of the patient and the high risk of sudden death caused by a giant left atrial mass obstructing the mitral valve. Intraoperatively, the tumor was solid and had a broad-based attachment to the left atrial endocardium. While the tumor was resected en bloc, intraoperative frozen section analysis could not be performed because of the emergency nature of nighttime surgery, and the definitive diagnosis was established via permanent pathological sections. As emphasized in various reports, the completeness of resection (R0 vs. R1/R2) significantly affects the median OS. Even after complete macroscopic resection, microscopic residual tumors may persist in the surrounding endocardial tissues. Therefore, intraoperative pathological confirmation is crucial.

Immunohistochemical analysis of 100 cardiac sarcomas revealed that the intimal sarcomas were 100% positive for MDM2, 71% positive for CDK4, and 88% positive for HMGA2. MDM2 amplification has been demonstrated exclusively in intimal sarcomas, making it a highly specific diagnostic marker.^10)^ In our case, the diagnosis of intimal sarcoma was confirmed based on the positivity for both MDM2 and CDK4.

Although macroscopic complete en bloc resection was achieved during the initial surgery, subsequent histopathological examination revealed the presence of atypical tumor cells within the endocardium at the site of broad tumor attachment. Based on this pathological evidence of microscopic residual disease, options for adjuvant chemotherapy were thoroughly evaluated. However, due to the lack of an established standard regimen for primary cardiac intimal sarcoma in Japan, combined with concerns regarding treatment-related toxicities, a consensus was reached among the medical oncologists, the patient, and his family to prioritize rigorous image-based surveillance with computed tomography and echocardiography over adjuvant treatment. Despite this intensive follow-up, a 1.5-cm recurrent tumor was detected 6 months postoperatively, which infiltrated the atriomitral junction and extended into the aortic annulus.

Considering the age of the patient and the aggressive nature of the malignancy, we opted for radical re-resection. Despite its high invasiveness, the commando procedure is used for oncological clearance. Originally described by David et al., it involves combined aortic and mitral valve operations with reconstruction of the intervalvular fibrosa.^11)^ This technique is primarily selected for tissue destruction caused by infective endocarditis, with variations such as “hemi-Commando” or “root-Commando” adapted to the extent of destruction.^12,13)^ However, reports of the Commando procedure being utilized to ensure radicality for recurrent primary cardiac malignancies are exceptionally rare.

Six months have passed since the second surgery (1 year after the initial surgery), and the patient remains free of recurrence. Continued rigorous follow-up is necessary to evaluate the long-term validity of this aggressive surgical strategy.

## Conclusion

For young patients with recurrent cardiac intimal sarcoma, the Commando procedure is a necessary strategy to achieve R0 resection despite its high surgical invasiveness. Achieving complete oncological resection through such aggressive reconstruction remains the only viable option to improve the prognosis of this otherwise fatal malignancy.

## Declarations

### Data availability

The data supporting the findings of this study are available from the corresponding author upon reasonable request.
